# Telehealth is Face-to-Face Service Delivery

**DOI:** 10.5195/ijt.2017.6225

**Published:** 2017-06-29

**Authors:** JANA CASON

**Affiliations:** SPALDING UNIVERSITY, LOUISVILLE, KY, USA

**Keywords:** Telehealth, Face-to-Face, In-person

## Abstract

The Commentary contests the increasingly outdated and narrow use of the terminology ‘face-to-face’ (often abbreviated as F2F) to connote clinical interactions in which both the client and the practitioner are physically present in the same room or space. An expanded definition is necessary because when delivered synchronously via videoconferencing, telehealth also provides face-to-face services (i.e., the practitioner and the client view each other’s faces). Terminology that uses face-to-face to connote only in-person care is limiting and perpetuates language that is out of line with progressive US regulatory language and broad interpretation within existing regulatory language. It is this author’s hope that this commentary will raise awareness of the important policy implications associated with this seemingly minor distinction in terminology and impact the lingering misapplication of the term, face-to-face.

The Commentary contests the increasingly outdated and narrow use of the terminology ‘face-to-face’ (often abbreviated as F2F) to connote clinical interactions in which both the client and the practitioner are physically present in the same room or space. An expanded definition is necessary because when delivered synchronously via videoconferencing, telehealth also provides face-to-face services (i.e., the practitioner and the client view each other’s faces). Terminology that uses face-to-face to connote only in-person care is limiting and perpetuates language that is out of line with progressive US regulatory language and broad interpretation within existing regulatory language. Therefore, the use of face-to-face should include telehealth applications (Cason, 2012).

## TELEHEALTH IS FACE-TO-FACE

In his 2011 State of the Union Address, President Barak Obama described the use of telehealth as being face-to-face (Linkous, 2011). President Obama stated: “It’s about a firefighter who can download the design of a burning building onto a handheld device; a student who can take classes with a digital textbook; or a patient who can have face-to-face video chats with her doctor” (Obama, 2011). Federal regulation supports the broad interpretation of face-to-face to include telehealth. For example, the Centers for Medicare & Medicaid Services (CMS; n.d.) defines telemedicine (used interchangeably in this commentary with telehealth) as “a cost-effective alternative to the more traditional face-to-face way of providing medical care (e.g., face-to-face consultations or examinations between provider and patient) that states can choose to cover under Medicaid” (para 2). Furthermore, CMS endorsed the use of telehealth to meet the face-to-face provision for Medicaid home health services (CMS, 2016).

State regulation also uses the term face-to-face when referencing telehealth. For example, the Arkansas Medical Board define a “proper physician-patient relationship” to include “a face-to-face examination using real time audio and visual telemedicine technology that provides information at least equal to such information as would have been obtained by an in-person examination” (Arkansas Medical Board, Section 8A1B). States have been responsive to the Centers for Medicare & Medicaid Services interpretation that telehealth services are face-to-face when delivered synchronously with audio and video. Several states have passed or introduced legislation expanding their definition of face-to-face services to include telehealth in regulatory language (Iowa Department of Human Services, 2017; Kentucky Cabinet for Health and Human Services, 2016; Minnesota State Legislature, 2017a; 2017b).

## IN-PERSON VS. FACE-TO-FACE

Arguably, the term face-to-face is commonly used in the literature and media when the intended meaning is ‘in-person’ care. This usage inherently implies that telehealth, via videoconferencing, is not face-to-face care. Researchers and other stakeholders are therefore encouraged to shift their terminology and use the term in-person rather than face-to-face when describing a traditional, in-person model of care in which all parties are present within the same physical space. This shift will improve the consistency of prevailing terminology and better align with progressive telehealth policy and regulatory language.

It is this author’s hope that this commentary will raise awareness of the important policy implications associated with this seemingly minor distinction in terminology and impact the lingering misapplication of the term, face-to-face.
